# Insights into the cavitation morphology of rubber reinforced with a nano-filler

**DOI:** 10.1038/s41598-023-33137-8

**Published:** 2023-04-10

**Authors:** Ryo Mashita, Yasumasa Bito, Kentaro Uesugi, Masato Hoshino, Ikuo Kageyuki, Hiroyuki Kishimoto, Wataru Yashiro, Toshiji Kanaya

**Affiliations:** 1 Chemical Analysis Center, Sumitomo Rubber Industries Ltd, Kobe, Japan; 2 Japan Synchrotron Radiation Research Institute, Hyogo, Japan; 3 International Center for Synchrotron Radiation Innovation Smart, Tohoku University, Sendai, Japan; 4 Kyoto University, Kyoto, Japan

**Keywords:** Soft materials, Structural materials

## Abstract

Notwithstanding the various uses of rubber, the fracture mechanism of filler-reinforced rubber remains unclear. This study used four-dimensional computed tomography (4D-CT) involving monochromatic synchrotron X-rays to examine the cavitation within silica-reinforced rubber quantitatively and systematically. The results suggested a threshold value of silica content for the cavitation morphology. Macroscopic fractures, such as those developed by void formation, occurred below the threshold value of silica content. Above this threshold, the density of rubber decreased but macroscopic voids rarely occurred. The lower-density rubber area in the high-silica-content rubber was reversible at the effective pixel size for 4D-CT. These results suggest that the growth of the damage points to macrosized voids could be stopped by the formation of a network of rigid polymer layers. This study allows the elucidation of the reinforcing mechanism and the cavitation morphology of filler-reinforced rubber.

## Introduction

Rubber is used in various commercial applications. Depending on the intended use, rubber must possess several properties, such as high mechanical strength and resistance to fracture, light, and deterioration. To realize a sustainable society via the conservation of natural resources, the mechanical strength and fracture resistance of rubber products must be improved. However, the fracture mechanism of rubber remains unknown because rubber materials are complex and consist of various additive agents. For example, nano-fillers, such as silica and carbon black, are added to the polymer matrices of rubber materials used in tire-based products to improve their fracture resistance. These reinforcing fillers disperse inhomogeneously and form complex hierarchical structures that range in size from nano- to milli-meters in the rubber materials^1^.

To better understand the fracture phenomenon of such materials, the relationship between the complex hierarchical structures and fracture phenomenon should be determined and discussed by changing the material factor systematically. The fracture resistance of rubber materials is drastically changed upon addition of nano-fillers, which suggests that the effect of nano-fillers on the fracture phenomenon must be elucidated.

Previously, several researchers have used various methods, such as electron microscopy^2–4^ and X-ray computed tomography (CT)^5–14^, to study the fracture mechanism of polymeric materials. Additionally, many studies have focused on the impact of reinforced fillers, such as silica and carbon black (CB), on the mechanical properties of rubber during deformation^15–18^ and fracturing^19,20^. These studies have revealed complex relationships between material properties and filler properties, including filler volume fraction, surface area, shape, and dispersibility. For instance, in natural rubber (NR) reinforced with CB, the existence of large CB agglomerates, which could be easily separated from the rubber matrix, led to a shorter fatigue life^21^. Moreover, it has been reported that the fatigue crack growth rate significantly decreased with the addition of organo-montmorillonite nanoparticles in NR reinforced with CB^22^. In styrene-butadiene rubber (SBR) reinforced with CB, agglomerates of metallic and other particle types, such as silica and zinc oxide, were observed at the bottom of well-developed cavities through damaged surface observation^5^. However, in NR reinforced with CB, the contribution of inherent particles, such as zinc oxide, to macroscopic crack initiation due to cavitation was found to be low^23^. Nano-voids were observed around silica aggregates using transmission electron microscopy (TEM) under deformation in isoprene rubber reinforced with CB^24^. Additionally, the fatigue life improved due to the addition of nano-dispersed clay into CB-filled SBR^25^. The researchers suggested that nano-dispersed clay layers could blunt the crack when distributed over CB. While several studies have investigated the effects of various fillers on the damage properties of rubber, the fundamental mechanism of the fracture phenomenon remains unclear.

Cavitation, which is a typical deformation-induced damage process, has been studied^26,27^. It may be induced by the constrained tension of a disk-shaped specimen, which is a sample with a thickness smaller than its diameter. The shortening of the sample length due to the Poisson effect is perpendicular to the stretching direction^4,28^. Thus, in this study, fracturing, that is, cavitation, was dominated by volume deformation. Additionally, the sizes and number of voids forming within the rubber depended on the diameter-to-thickness ratio of the disk-shaped sample^4,6^. The dependence of the number and shape of voids on the type of polymer^5^ and the effect of reinforcing fillers on the volume and formation process of voids^6,7^ were analyzed. However, the effect of filler reinforcement on the cavitation of rubber has not been completely elucidated. The cavitation that appear inside rubber materials can be observed via X-ray CT because X-rays can penetrate the rubber material. This is partly because the obtained CT images cannot be quantitatively analyzed by CT methods that utilize white X-rays in a standard laboratory analyzer as the white X-rays contain several energy levels. Thus, it is difficult to accurately measure the change in rubber density during the cavitation process by using white X-rays. As an alternative to CT methods, four-dimensional CT (4D-CT) that utilizes monochromatic X-rays has been performed at synchrotron radiation facilities. We have previously observed the cavitation in rubber using the synchrotron X-ray 4D-CT method, which allowed us to successfully determine the growth behavior of voids^8,9^. The change in rubber density during the cavitation process must also be measured and the effect of reinforcing fillers on this change must be elucidated.

The novelty of this study lies in the observation that rubber exhibits two types of cavitation morphology depending on the amount of the reinforcing filler. In this study, the effect of the amount of the reinforcing filler on the cavitation in rubber is investigated quantitatively and systematically using the 4D-CT method that utilizes monochromatic synchrotron X-rays. The results of this study are expected to provide deeper insights into the mechanism of filler reinforcement and the cavitation process of rubber toward the larger goal of realizing a sustainable society via conservation of natural resources.

## Experimental

### Materials and sample preparation

Styrene-butadiene rubber (SBR) filled with various amounts of a reinforcing nano-filler, in this case silica, was used as the sample. Silica is typically utilized in passenger car tires for daily use, and thus, it was used as a reinforcing filler in this study owing to its industrial applications. The details of the materials used in this study are summarized in Table 1. Five samples containing various amounts of silica were prepared: Si(0), Si(0.1), Si(0.15), Si(0.2), and Si(0.25). In the sample labels, the numbers within the parentheses refer to the volume fraction of silica (*Φ*) in the rubber sample. The average diameter of the primary silica particles used in this study was ~17 nm.

**Table 1:** Sample composition wherein the weight ratios were normalized using styrene-butadiene rubber.

	Si(0)	Si(0.1)	Si(0.15)	Si(0.2)	Si(0.25)
SBR^a^	100	100	100	100	100
Silica^b^	0	25.8	41.4	59.5	80.3
Silane coupling agent^c^	0	2.1	3.3	4.8	6.4
Stearic acid	2	2	2	2	2
Sulfur	1.3	1.3	1.3	1.3	1.3
Accelerator I^d^	1.3	1.3	1.3	1.3	1.3
Accelerator II^e^	0	2	2	2	2

^a^Styrene-butadiene rubber (styrene and vinyl butadiene contents are 26 and 44 wt.%, respectively).

^b^VN3 grade.

^c^4,4,13,13-Tetraethoxy-3,14-dioxa-8,9-dithia-4,13-disilahexadecane.

^d^N-Cyclohexyl-2-benzothiazole sulfonamide.

^e^1,3-Diphenylguanidine.

The raw materials listed in Table 1 were mixed in an internal mixer until the temperature of the compound reached 423 K. Next, the mixture was placed in a mold and heated at 443 K for 20 min to increase the rate of the crosslinking reaction. Consequently, a crosslinked rubber sheet was obtained. The sample sheets were glued between two brass sample holders using glue to form the required rubber samples for the 4D-CT measurements, which were cylindrical with a 20-mm diameter and a 1-mm height. According to the approach reported by Gent et al.^4,27,28^, the shape factor of the sample, *S*, is defined as $S=\pi {R}^{2}/(2\pi Rh)=R/(2h)$, where *R* and *h* are the radius and thickness of the sample, respectively. *S* = 5 in this study, suggesting that the shape of the sample is suitable for use in analyzing the fracturing of rubber materials because numerous voids are formed under the strong confinement of volume deformation (Poisson’s ratio, *ν* ≠ 0.5).

### Measurements

**Figure 1:**
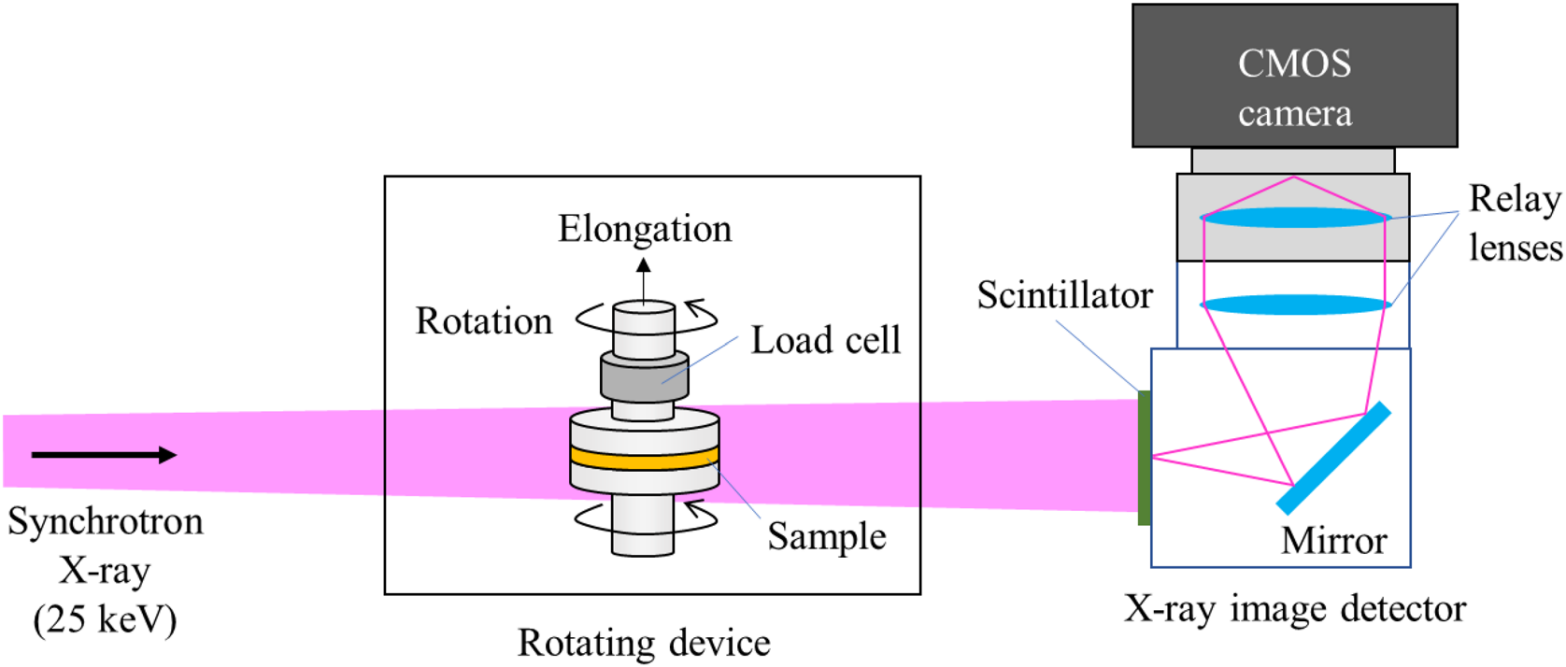
Experimental setup for X-ray tomography conducted using a rotating device at BL20B2 of SPring-8 (side view).

The cavitation phenomenon of rubber under deformation was observed using the 4D-CT method. The 4D-CT experiments were performed using BL20B2 at SPring-8, Japan, where a monochromatic synchrotron X-ray beam from a bending-magnet source was available. The incident X-ray energy, exposure time, and number of projections were 25 keV, 5 ms/projection, and 900, respectively. The dimensions of the field of view were 26.4 mm (horizontal) × 3 mm (vertical). The measured effective pixel size was 13.2 μm. Figure 1 shows the experimental setup for the 4D-CT experiment. A rotating device was used, in which the sample could be rotated with stretching. The load cell was installed in this rotating device, and the load applied to the sample was measured during deformation. The full experimental details are reported elsewhere^8^. The rubber sample was stretched along its height at a tensile rate of 0.44 μm/s. 4D-CT was continuously conducted during the elongation of the sample.

Dynamic viscoelasticity measurements were conducted using a dynamic mechanical analyzer at 303 K and 10 Hz to determine the elastic modulus.

Scanning electron microscopy (SEM) was performed at an operation voltage of 1 kV to observe the dispersion of silica within the rubber.

## Results

### 4D-CT observation of rubbers containing different amount of silica

**Figure 2:**
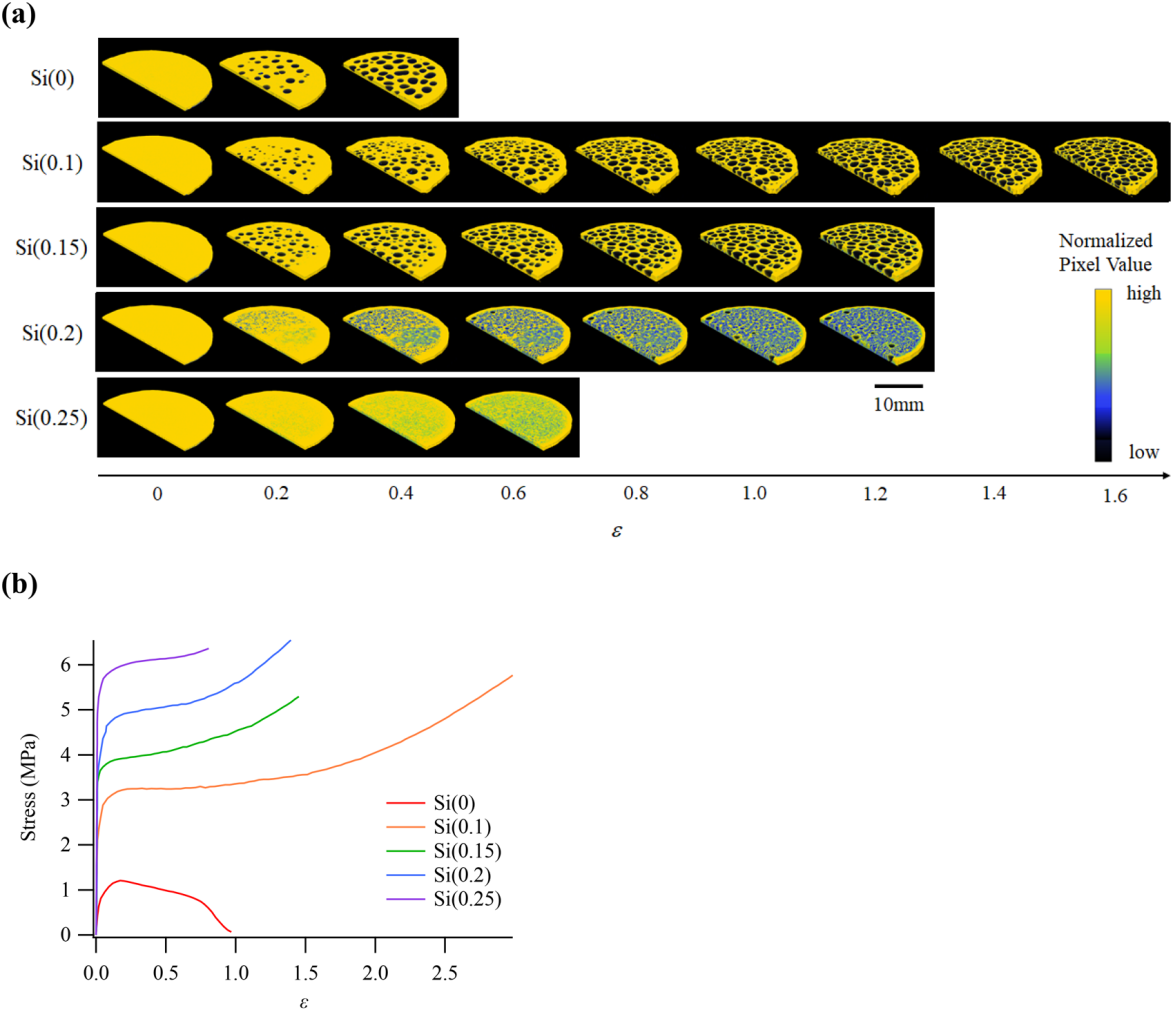
(**a**) Strain dependence of the three-dimensional images of each sample. (**b**) Stress–strain curves obtained during 4D-CT.

Figure 2 shows the 4D-CT results of the cavitation of rubber under deformation. Each scan was performed after the sample was rotated 180° and was obtained by applying the convolution back projection algorithm^29^ to the minus logarithm of the transmittance images. Figure 2a shows the 3D images of each rubber sample at a strain of $\varepsilon =\left(h-{h}_{0}\right)/{h}_{0}$, where *h* and *h*_0_ are the thicknesses of the sample after and before stretching, respectively. The tops of these 3D images show a middle slice of the rubber. Since the 3D images are also sliced along the direction of thickness of the rubber sample, their tops are semicircular. The yellow and black colors in the images represent higher- and lower-intensity pixels, respectively. The pixel intensity reflects the density of rubber because monochromatic X-rays were used. In principle, the density of rubber can be calculated from the X-ray absorption rate. However, it is difficult to calculate it accurately because of the cylindrical shape of the rubber sample. Therefore, the pixel intensity of rubber after stretching (*ε* > 0) was normalized with the average value of the pixel intensity of rubber before stretching (*ε* = 0). In other words, the yellow area corresponds to the area that is equal to the density of rubber before stretching. The black area, which appears inside the rubber at *ε* > 0, corresponds to the void, representing the completely fractured rubber part. The blue and green areas, which are called the lower-density rubber areas in this study, represent a middle state between the yellow and black areas.

In Fig. 2a, it is evident that the voids mainly appear inside the rubber at *Φ* < 0.15. At *Φ* ≥ 0.2, the lower-density rubber was mainly present, instead of the void. Therefore, it was concluded that there was a threshold value of silica content for the cavitation morphology, indicating that macroscopic fracture via void formation occurred below the threshold value of silica content. Above the threshold silica content, the rubber density decreased and macroscopic voids rarely occurred.

Figure 2b shows the stress–strain curves obtained using 4D-CT. In each sample, the stress increased with increasing strain *ε*. The slope of the stress increment plateaued at strains of 0.1–0.2. At these strain values, a gradual increase in stress is observed, and voids or lower-density rubber areas begin to form, resulting in stress changes that cause rubber deterioration.

Generally, stress increases gradually with increasing strain owing to the disentanglement of polymer chains in the simple tensile mode of uniaxial elongation without any volume change (i.e., Poisson’s ratio *ν* = 0.5)^30^. It is believed that the tensile mode of this study is accompanied with a volume change (*ν* < 0.5), resulting in a gradual increase in stress at strains of 0.1–0.2 because of the formation of voids or lower-density rubber areas.

To understand the dependence of the cavitation morphology on the silica content quantitatively, as shown in Fig. 2a, the change in rubber density was estimated by measuring the change in the pixel intensity of the CT images. Although the samples used in this study were mixtures of several types of materials dispersed inhomogeneously on the nanometer scale^1^, the change in rubber density can still be estimated from the change in the pixel intensity of the CT images because the composition of the samples can be considered homogeneous at an effective pixel size of 13.2 μm.

**Figure 3:**
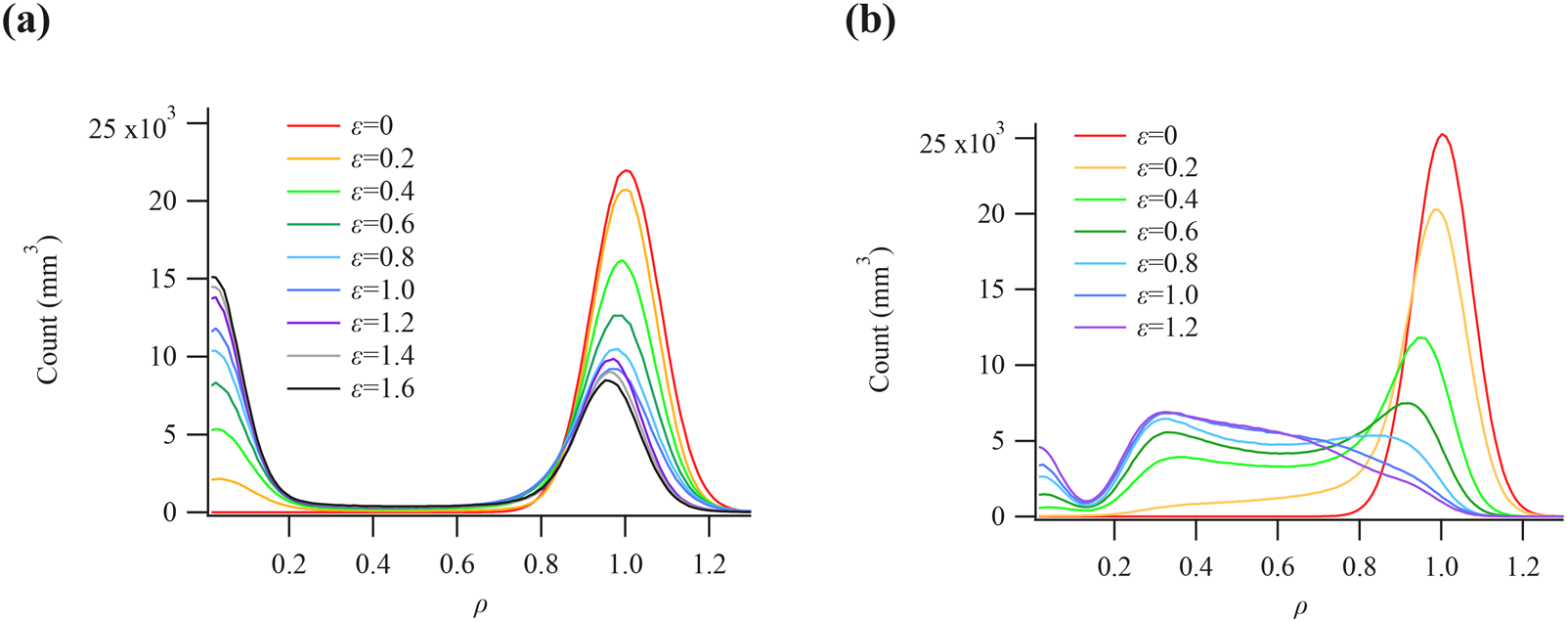
Histograms of the change in rubber density *ρ* calculated from computed tomography images of (**a**) Si(0.1) and (**b**) Si(0.2) at each strain *ε*.

Figure 3 shows the strain dependence of the histogram of density change per unit volume (*ρ*) of Si(0.1) and Si(0.2). The density change inside the cylindrical sample was analyzed at a distance of 17 mm from the center of the sample and a height of 0.26 mm from the base in order to investigate the cavitation morphology of only the internal rubber. Although a sharp peak centered at *ρ* = 1 could be seen before deformation (*ε* = 0) because the composition of the sample can be considered homogeneous (as previously mentioned), a broad peak, which had a full width at half maximum (FWHM) of ∼0.17, was seen in the obtained CT images. This result is attributed to the statistical error of incident photons or the readout noise of the detector. Therefore, the value of *ρ* in Fig. 3 includes the error of 0.17.

As shown in Fig. 3a, the intensity of the peak originating from rubber at *ρ* ≈ 1 decreased with increasing strain. Conversely, the intensity of the peak originating from the void at *ρ* ≈ 0 increased with increasing strain. Similar trends were observed for Si(0) and Si(0.15). It was determined that voids primarily formed inside the rubber sample at *Φ* < 0.15. In contrast, as shown in Fig. 3b, the intensity of the peak originating from the rubber at *ρ* ≈ 1 decreased with increasing strain, whereas that at *ρ* = 0.2–0.8 increased. The intensity of the peak that originated from the void at *ρ* ≈ 0 increased with increasing strain. The difference between the results shown in Fig. 3a,b is that the lower-density rubber area of 0.2 ≤ *ρ* ≤ 0.8 increased with increasing strain only for Si(0.2). The same trend as Si(0.2) was also observed for Si(0.25). These results reveal that the lower-density rubber area was only formed in Si(0.2) and Si(0.25), and that the macroscopic fracture, particularly via void formation, only occurred under specific conditions.

Comparisons of cavitation morphologies in rubber samples with varying filler amounts have been reported^10,31,32^, with a primary focus on the analysis of the size and number of cavities formed in the rubber. The cavity size in NR reinforced with CB was smaller for samples with high filler content than those with low filler content^10^. The same trend was observed in SBR reinforced with CB^31^, revealing that the number and size of cavities increased with strain, and as CB content increased, cavity size decreased while their number increased. In these studies, the terms “void” and “lower-density rubber area” were used interchangeably with “cavity.” The 4D-CT results confirmed that higher silica content led to smaller void sizes and lower-density rubber areas, as seen in Fig. 2a, consistent with previous findings. Moreover, the yield stress in rubber with high filler content was higher than that in rubber with low filler content^10^, which aligns with our results shown in Fig. 2b. The previous study concluded that cavities initiate from microbubbles or micro defects and grow independently in rubber with low filler content, while high filler concentration leads to large agglomerates, creating high local stress concentrations and multiple microcracks. Cavity formation in unfilled SBR and SBR filled with CB has also been reported^32^. The volume fraction of CB was estimated to be approximately 0.2 based on the sample contents. Only voids were observed in unfilled rubber, while areas presumed to be lower-density rubber were found in filled rubber. This observation is consistent with the results shown in Fig. 2a. Additionally, it was revealed for the first time in this study that the cavitation morphology changed after the threshold of silica content was exceeded because of the accurate analysis of the change in rubber density using monochromatic synchrotron X-rays.

### Properties of two types of cavitation morphologies

**Figure 4:**
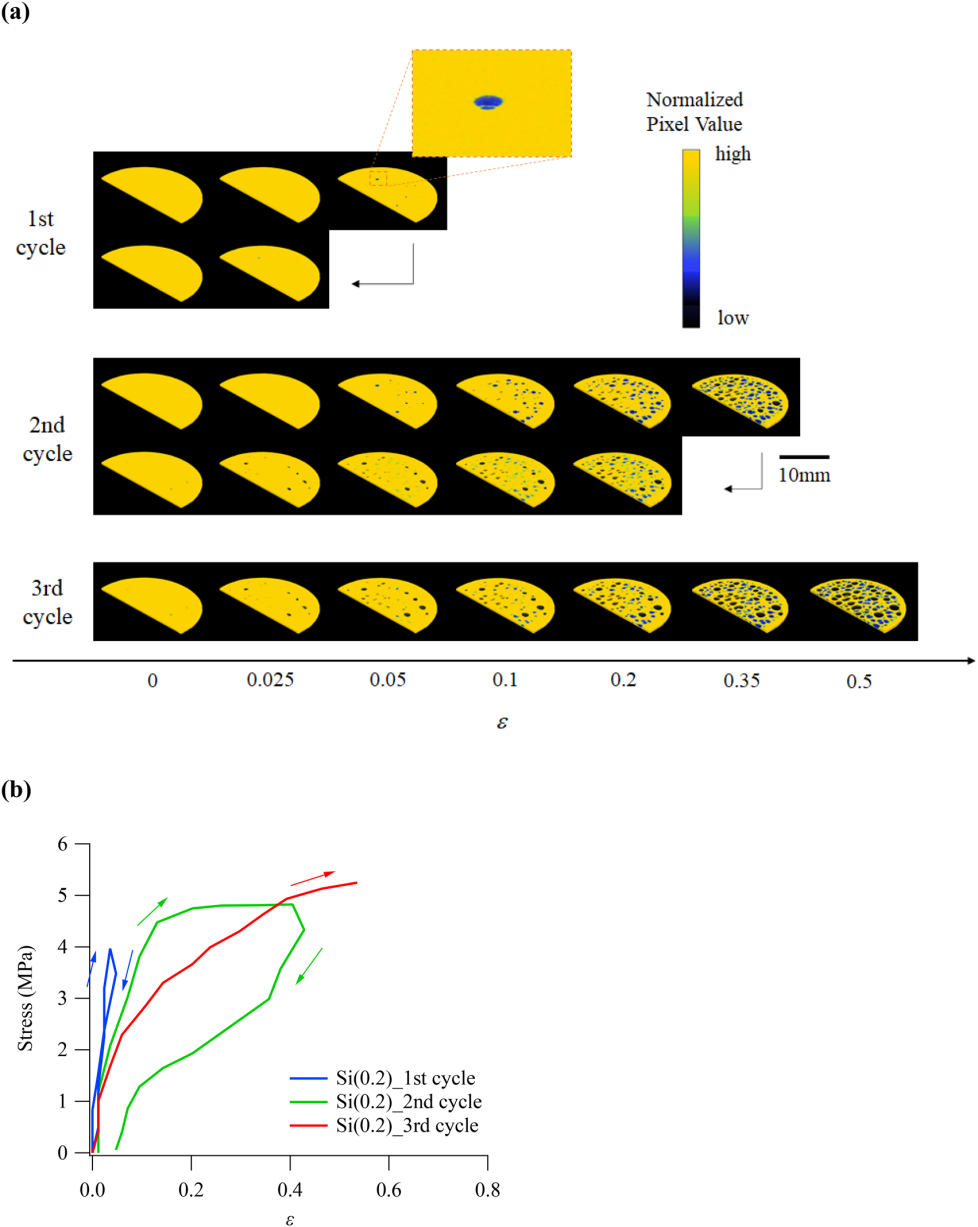
(**a**) Strain dependence of three-dimensional images under repeated deformation in Si(0.2). (**b**) Stress–strain curves obtained on the basis of 4D-CT results. The blue, green, and red lines represent the first, second, and third cycles of repeated deformation, respectively.

To elucidate the properties of the voids and lower-density rubber areas seen in Figs. 2 and 3, the cavitation behavior of Si(0.2) was observed under repeated deformation. Figure 4a and Movie S1 (Supporting Information) show the 4D-CT results of the cavitation in rubber under repeated deformation. The stress–strain curves obtained under repeated deformation are shown in Fig. 4b. The method used to apply the repeated deformation was as follows. In the first cycle, the sample was stretched at *ε* = 0.05. Immediately after, it was shortened at *ε* = 0. In the second cycle, it was stretched again at *ε* = 0.4. Again, immediately after, it was shortened at *ε* = 0. In the third cycle, it was stretched until it was broken. Movie S1 shows every *ε* = 0.025 in the first, second, and third cycles.

As shown in Fig. 4a and Movie S1, only the lower-density rubber area appeared with stretching and then disappeared with shortening until *ε* = 0 in the first cycle. In the second cycle, the lower-density rubber area first appeared at the same point where it occurred in the first cycle. Then, the voids formed at *ε* ≈ 0.2 with stretching. It was also observed in the second cycle that the lower-density rubber area disappeared with shortening, followed by the disappearance of the voids after a delay. In the third cycle, the voids were first formed at a very low strain at *ε* = 0.01, followed by the appearance of the lower-density rubber area, which is contradictory to the observation in the first and second cycles. These results indicate that the lower-density rubber areas are reversible at an effective pixel size during 4D-CT and that voids cannot return to their original state once they are formed.

The reversibility of the formation of lower-density rubber areas is evident from the fact that the voids appeared at the same point in the first and second cycles. The voids seem to possess a degree of reversibility because they were formed upon stretching and then disappeared with shortening in the second cycle. However, they ultimately disappeared because the voids, i.e. the rubber damage, which occurred during the stretching process, were compressed owing to the contact between the fracture surfaces during the shortening process. The non-reversibility of the void formation is evident from the fact that they appeared at the lowest strain before the appearance of the lower-density rubber area in the third cycle, indicating that the void formed in the second cycle did not disappear and that the compressed void was opened with the slightest stretching. Moreover, a thin void was observed at *ε* = 0 at the beginning of the third cycle. It is theorized that a slight gap occurred because the fracture surfaces were not completely in contact with each other owing to the compressed voids that were formed in the second cycle. In addition, the strain does not return to its initial value in the second cycle, and thus, non-reversible void formation occurs and the rubber deforms plastically. These results also support the conclusion that void formation is non-reversible. It was revealed for the first time that rubber exhibits two types of cavitation morphologies, viz. a reversible lower-density rubber area and a non-reversible void.

## Discussions

### Properties of the lower-density rubber area

Although microscopic voids were not clearly observed in the lower-density rubber area at the effective pixel size of this study (13.2 μm), tiny satellite cavities were identified in the walls of neighboring microscopic cavities using micro-CT with a higher spatial resolution^31^. In addition, nanovoids were revealed using in situ small-angle X-ray scattering^33^ and transmission electron microscopy^24^ under deformation. In the case of silica with weak interfacial interactions, fibril-like cavitation was observed around the silica surface^34^. Conversely, in the case of silica with strong interfacial interactions, cavitation was observed within the rubber matrix. Similar results have been obtained via molecular dynamics simulations^35–37^. When the nanoparticle-polymer interaction is attractive, nanovoids are observed in the polymer bulk, whereas when it is repulsive, nanovoids are formed at the interface between the polymers and nanoparticles^35^. Based on these results, fracturing could occur in the lower-density rubber area on a scale smaller than the effective pixel size of 4D-CT (13.2 μm).

### Relationship between the cavitation morphology and formation of a rigid network

**Figure 5:**
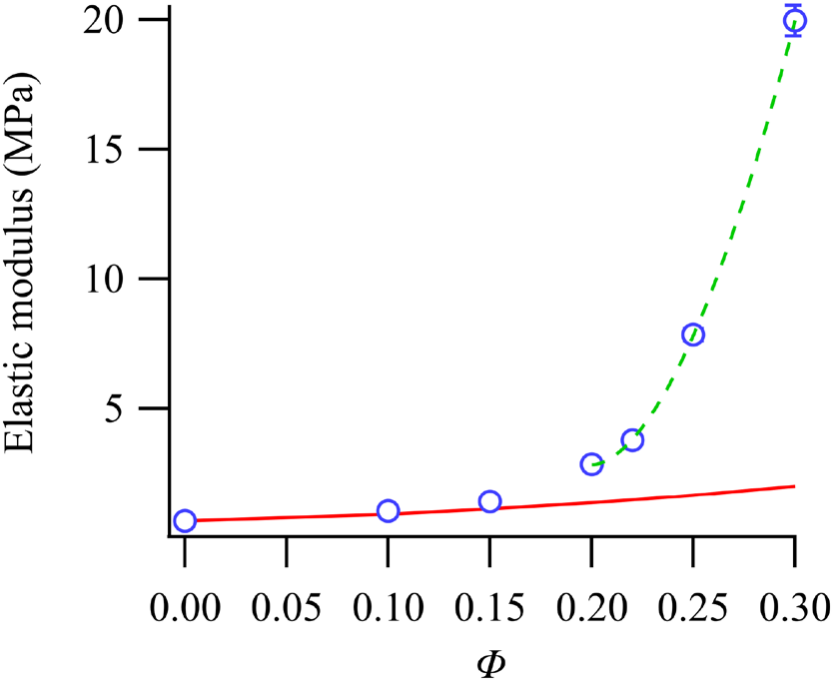
Relationship between the elastic modulus and *Φ*. The solid and dotted curves represent the fitting results of the Guth–Gold Eq. (1) and percolation theory Eq. (2), respectively.

The elastic modulus of rubber and the dispersion state of silica were measured to elucidate the silica content dependence of the cavitation morphology observed through 4D-CT results. Figure 5 shows the relationship between the elastic modulus and *Φ*. The elastic modulus increased gradually with increasing *Φ* before increasing drastically after *Φ* = 0.15. The Guth–Gold equation, as shown in Eq. (1), is generally used to discuss the filler content dependence of the elastic modulus of rubber-containing fillers^38,39^.

1 $$E/{E}_{0}=1+2.5\Phi +14.1{\Phi }^{2}$$

where *E*_0_ and *E* represent the elastic moduli of the unfilled and filled rubber, respectively. Although the experimental and calculated elastic moduli were in good agreement at low *Φ*, the experimental value was greater than the calculated value at *Φ* > 0.2, as seen in Fig. 5. Thus, the effective volume fraction of the filler was larger than the filler content added to the rubber because of the bound rubber enclosed by aggregated nano-fillers, such as silica and carbon black, and the rigid polymers absorbed on the surfaces of the nano-fillers^40,41^. In addition, the elastic modulus was thought to increase owing to the network formation of rigid polymers confined within the fillers. The presence of this confined rigid polymer layer was confirmed by various methods, including atomic force microscopy^42^, nuclear magnet resonance spectroscopy^43^, and the small-angle neutron scattering^44–46^ and quasielastic neutron scattering^47^ techniques.

Although the modified Guth–Gold equations, considered for the confined rigid polymer layers, were suggested^48,49^, the filler content dependence of the elastic modulus was not completely explained by these equations because it was difficult to accurately measure the elastic modulus of the confined rigid polymer layer and the degree of network development. It was also considered that the rigid polymer layer was formed on the surface of silica because silica and the polymers were bonded chemically by using a silane coupling agent. Thus, it is indicated that such rigid polymer layers increased along with *Φ*. Furthermore, the rigid polymer network was formed owing to the connection of such layers. A notable result was that the experimental *Φ* range of the elastic modulus (Fig. 5) differed from that calculated using Eq. (1) in Fig. 5; however, it corresponded well with the *Φ* range at which the cavitation morphology changed in Fig. 2a (*Φ* ≥ 0.2). This indicates that the difference in the cavitation morphology is related to the formation of the rigid polymer layer network confined on the silica surface.

**Figure 6:**
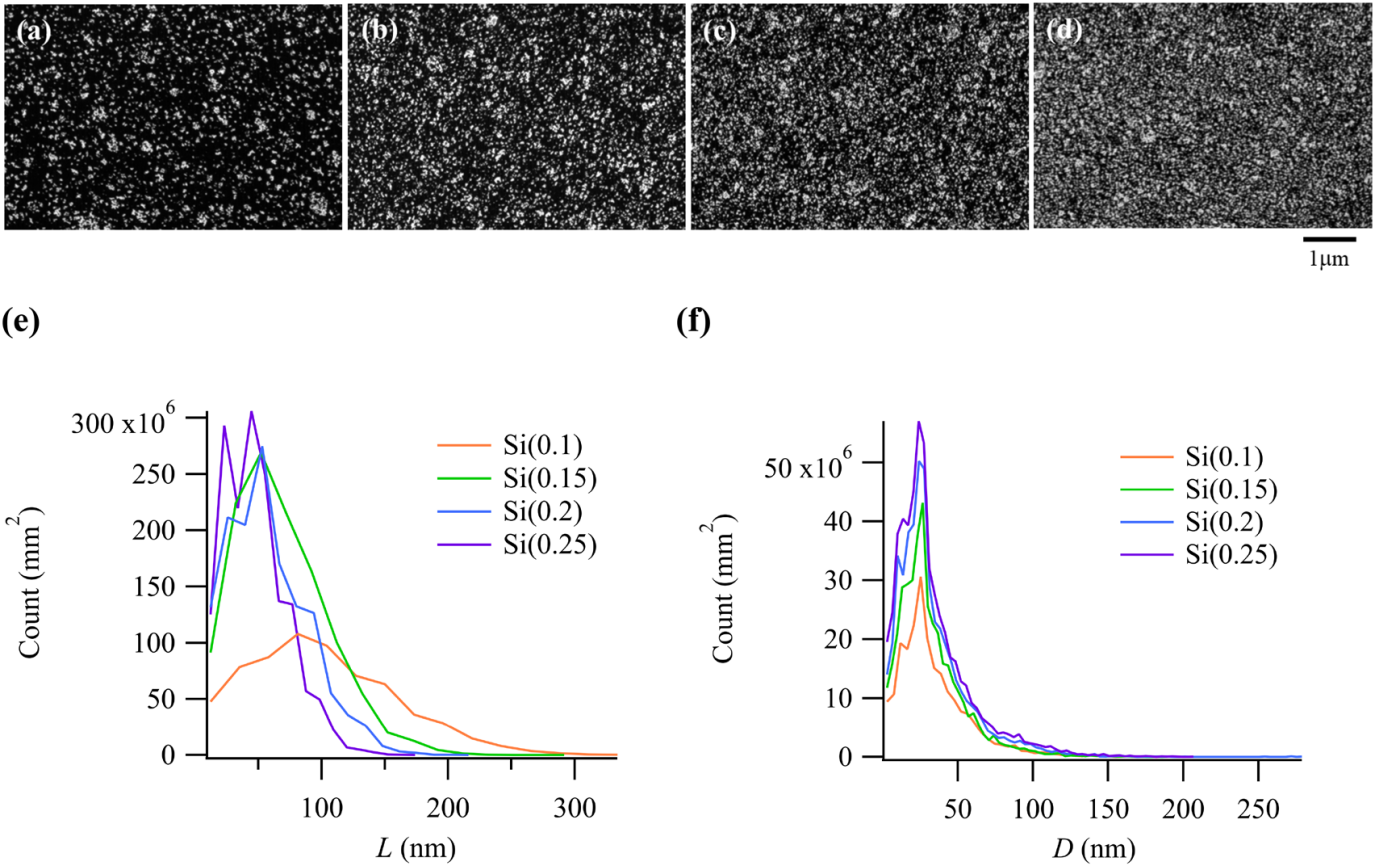
Scanning electron microscopy (SEM) images of (**a**) Si(0.1), (**b**) Si(0.15), (**c**) Si (0.2), and (**d**) Si(0.25). Histograms of (**e**) surface-to-surface silica distance *L* and (**f**) diameter of the silica aggregate *D*, obtained from the SEM images.

The dispersion state of silica was observed by scanning electron microscopy (SEM) to determine the relationship between the formation of the rigid polymer network and the percolation of silica. Figure 6a–d show the SEM images of Si(0.1) − Si(0.25), respectively, while Fig. 6e,f show the histograms of the surface-to-surface silica distance (*L*) and the diameter of the silica aggregate (*D*), respectively. *L* was calculated from the SEM images using the Voronoi tessellation method^50^. The mean values of *L* and *D* are summarized in Table 2. Parameter *D* had a nearly constant value independent of *Φ*, whereas *L* decreased with an increase in *Φ*. Thus, it is theorized that the surface-to-surface silica distance decreased with increasing *Φ* and that the rigid polymer layers around silica were connected, resulting in the formation of a rigid polymer network.

**Table 2:** Mean values of the surface-to-surface silica distance *L* and diameter of the silica aggregates *D.*

	Si(0.1)	Si(0.15)	Si(0.2)	Si(0.25)
Mean value of *L* (nm)	112	77	64	53
Mean value of *D* (nm)	36	34	35	35

The relationship among various properties, such as elastic modulus and conductivity, and the correlation between the material structure and the percolation theory have been extensively analyzed^51–55^. The percolation theory was applied to our system using Eq. (2):

2 $$E/{E}_{0}\propto {\left({\Phi }_{eff} - {\Phi }_{eff, c}\right)}^{n},$$

where ${\Phi }_{eff}$ and ${\Phi }_{eff, c}$ are the effective volume fractions of silica (viz. the rigid polymer layer and critical percolation value, respectively) and *n* is the scaling exponent. The thickness of the rigid polymer layer must be known to calculate ${\Phi }_{eff}$. Takenaka et al*.* determined the thickness of the absorbed polymer layer around the silica surface to be 5.3 nm in a solvent using the contrast-variation small-angle neutron scattering method in a sample system similar to that used in this study^44^.

Based on the similarity between the polymer and silane coupling agent types, it is expected that the thickness of the rigid polymer layer in our system would be similar to that in the afore-mentioned study^44^. Thus, the thickness of the rigid polymer layer in our system was assumed to be 5 nm. As seen in Table 2, the mean value of the silica aggregate diameter was ∼35 nm; consequently, ${\Phi }_{eff}$ was estimated based on the assumption that the 5-nm-thick rigid polymer layer existed around a spherical silica aggregate with a 35-nm diameter. Thus, ${\Phi }_{eff, Si(0.15)}$ = 0.32 and ${\Phi }_{eff, Si(0.2)}$ = 0.43 were obtained for Si(0.15) and Si(0.2), respectively.

Next, we compared the calculated values and the ${\Phi }_{eff, c}$ obtained by fitting the *Φ* dependence of the elastic modulus with Eq. (2), as shown in Fig. 5. By applying the calculated values from Eq. (2), ${\Phi }_{eff, c}$ = 0.42 and *n* = 1.78. The fact that ${\Phi }_{eff, Si(0.15)}$ < ${\Phi }_{eff, c}$ indicates that the rigid polymer network is not formed in Si(0.15). Moreover, because ${\Phi }_{eff, Si(0.2)}$ > ${\Phi }_{eff, c}$, it is indicated that the rigid polymer network is formed in Si(0.2); these results are consistent with those derived from the network formation in terms of the deviation between the *Φ* dependence of the elastic modulus and Eq. (1). It was also reported that the scaling exponent (*n*) was generally 1.2–2.0 for the conductivity and elastic modulus^51,52,56^. The value of *n* (= 1.78) obtained from fitting Eq. (2) was within this range, indicating consistency with the results of previous reports. Although the estimate is based on several assumptions, the results were supported by the percolation theory that the rigid network was formed owing to the rigid polymer layer on the surface of silica at *Φ* ≥ 0.2.

Based on these results, the cavitation morphology of the rubber reinforced with silica was elucidated. The points of origin of the cavitation occur inside the rubber, and such points expand upon elongation. When the silica content is low, the growth of the damage points cannot be stopped owing to the absence of rigid polymer layer network formation, resulting in the growth of them into macrosized voids larger than the effective pixel size (13.2 μm) for 4D-CT. Conversely, when the silica content is high, the growth of the damage points to the macrosized voids can be stopped by the formation of a rigid polymer layer network. It was suggested that the lower-density rubber area, as formed in Si(0.2) and Si(0.25) (Fig. 2a), occurred as a result of controlling the growth of the damage point to less than the effective pixel size for 4D-CT because of the formation of the rigid polymer layer network. In conclusion, it was revealed that rubber has two types of cavitation morphologies, viz. a non-reversible void and reversible lower-density rubber area, and that the cavitation morphology of rubber depended on the silica content, through a detailed analysis of the density changes occurring inside the rubber under deformation by conducting 4D-CT.

## Conclusion

We quantitatively and systematically studied the cavitation in the rubber reinforced with silica via 4D-CT conducted using monochromatic synchrotron X-rays. The objective of this study was to determine the effect of the amount of the reinforcing filler on the cavitation morphology under deformation.

It was observed that the voids mainly appear inside the rubber at *Φ* < 0.15. At *Φ* ≥ 0.2, the lower-density rubber area mainly appeared instead of the void. Therefore, it was elucidated that there was a threshold value of silica content for the cavitation morphology, indicating that the macroscopic fracture (via void formation) occurred below the threshold value of silica content. Above the threshold value of silica content, the rubber density decreased and the macroscopic voids rarely occurred. In addition, to elucidate the property of the void and lower-density rubber area, the cavitation behavior under repeated deformation was observed. It was revealed that the lower-density rubber area is reversible at the effective spatial resolution for 4D-CT and the void cannot return to its original state of rubber once it is formed.

It was also determined that the range of *Φ* over which the experimental results of the elastic modulus differed from those calculated by the Guth–Gold equation corresponded with that of *Φ* over which the cavitation morphology changed in the 4D-CT observations (*Φ* ≥ 0.2). This result indicates that the difference in the cavitation morphology is related to the formation of the rigid polymer layer network confined on the silica surface. The dispersion state of silica was observed through SEM in order to reveal the relationship between the formation of the rigid polymer network and the percolation of silica. *D* showed a nearly constant value regardless of *Φ*, while *L* shortened with an increase in *Φ*. Thus, it is expected that *L* decreased along with *Φ* and that the rigid polymer layers around silica were connected, resulting in the formation of a rigid polymer network. This hypothesis was supported by the percolation theory, in which it is assumed that the 5-nm-thick rigid polymer layer exists around the spherical silica aggregate.

In this study, it was revealed that rubber has two types of cavitation morphologies, namely, a non-reversible void and reversible lower-density rubber area. When the silica content was low, the growth of the damage points could not be stopped because no rigid polymer layer network was formed, which resulted in the considerable growth of them, such as to the non-reversible macrosized voids, which are larger than the effective spatial resolution for 4D-CT. When the silica content was high, the growth of the damage point to the macrosized void could be stopped because of the formation of a rigid polymer layer network, which resulted in the creation of a reversible lower-density rubber area.

## Supplementary Information

Supplementary Information 1.

Supplementary Video 1.

## Data Availability

All the data of this study are available from the corresponding author upon reasonable request.
