# Supplementary material for: Insights into the cavitation morphology of rubber reinforced with a nano-filler
Source: Sci Rep. 2023 Apr 10;13:5805. doi: 10.1038/s41598-023-33137-8 (PMC10086055; doi:10.1038/s41598-023-33137-8)
Supplement: Supplementary file 1 — Supplementary Information 1. [file 41598_2023_33137_MOESM1_ESM.docx]

Supplementary Movie S1

4D-CT result of Si(0.2) under repeated deformation.
